# Mass spectrometric insights into protein aggregation

**DOI:** 10.1042/EBC20220103

**Published:** 2023-03-29

**Authors:** Tara L. Pukala

**Affiliations:** Department of Chemistry, School of Physical Sciences, University of Adelaide, Adelaide, South Australia 5000, Australia

**Keywords:** mass spectrometry, protein aggregation, protein structure

## Abstract

Protein aggregation is now recognized as a generic and significant component of the protein energy landscape. Occurring through a complex and dynamic pathway of structural interconversion, the assembly of misfolded proteins to form soluble oligomers and insoluble aggregates remains a challenging topic of study, both *in vitro* and *in vivo*. Since the etiology of numerous human diseases has been associated with protein aggregation, and it has become a field of increasing importance in the biopharmaceutical industry, the biophysical characterization of protein misfolded states and their aggregation mechanisms continues to receive increased attention. Mass spectrometry (MS) has firmly established itself as a powerful analytical tool capable of both detection and characterization of proteins at all levels of structure. Given inherent advantages of biological MS, including high sensitivity, rapid timescales of analysis, and the ability to distinguish individual components from complex mixtures with unrivalled specificity, it has found widespread use in the study of protein aggregation, importantly, where traditional structural biology approaches are often not amenable. The present review aims to provide a brief overview of selected MS-based approaches that can provide a range of biophysical descriptors associated with protein conformation and the aggregation pathway. Recent examples highlight where this technology has provided unique structural and mechanistic understanding of protein aggregation.

## Significance of protein aggregation

Proteins rely on a high degree of conformational flexibility to fold into their native states and facilitate the extensive interactions with a wide variety of binding partners that underlie biological function. Reaching and maintaining such native structures is enabled by a downhill-folding pathway on a free-energy surface. However, the near cancellation of enthalpic and entropic contributions to free energy during the folding process, and the low energy barriers to structural interconversion, mean that the flexibility required for a protein to function often places them at the brink of stability [1]. Protein aggregation is therefore increasingly considered as a fundamental aspect of the protein conformational landscape [2,3], and is a particularly widespread phenomenon in the cellular environment [4–7].

**Figure 1:**
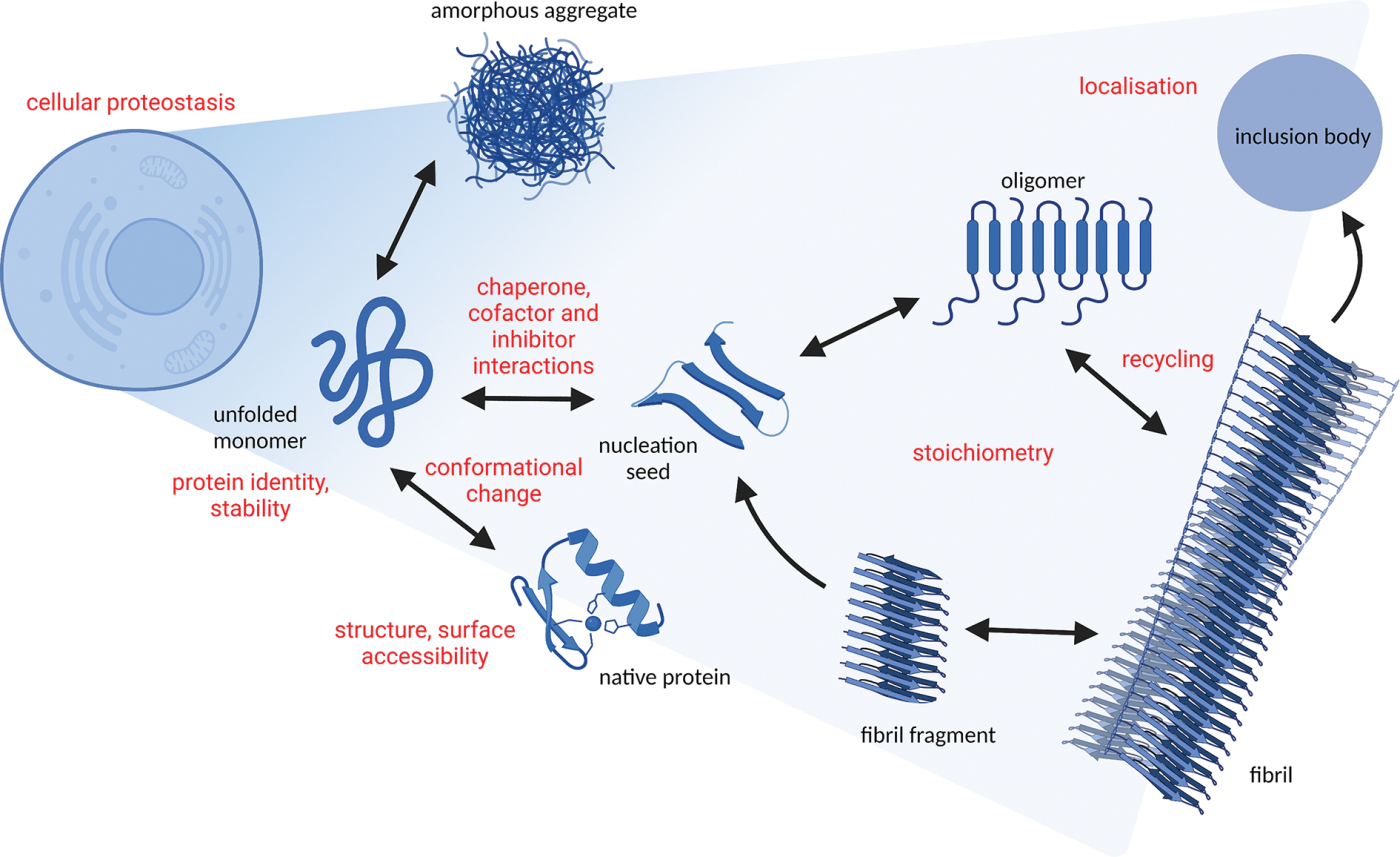
Insights from mass spectrometry (MS) on the pathway of protein aggregation An ensemble of possible structures exist for any given protein, whereby non-native conformations are prone to associate and form aggregates. Nonfibrillar (amorphous) aggregates result from nonspecific interactions between many different conformations. In contrast, association of soluble species (usually monomers) into β-sheet-rich intermediates gives rise to an aggregation nucleus wherein a growing pool of soluble oligomers triggers polymerization and fibrillization. MS-based approaches can provide important biophysical insight at multiple stages of the aggregation pathway, as highlighted in red text.

Protein aggregation refers to the multistep process by which abnormal association of proteins gives rise to larger aggregate structures, which tend to be insoluble. This process commences with the self-assembly of misfolded monomers to produce a range of intermediates termed soluble-misfolded oligomers. Further assembly from such early oligomeric states leads to formation of insoluble structures in the forms of amorphous aggregates or amyloid fibrils with ordered β-sheet conformations [1,8] (Figure 1). This can occur under both normal physiological conditions and in response to age or stress-induced protein misfolding and denaturation. For example, a wide range of environmental conditions, such as changes in pH, temperature or oxidative stress, disruption to heavy metal homeostasis, and other modifications, can shift the protein conformational equilibrium toward more aggregation prone states [9]. Understanding this process is a central issue in many fields of biology and biotechnology, especially as many disease states are associated with this phenomenon. In particular, the aberrant deposition of neuronal protein aggregates as amyloid fibrils is a common pathological hallmark of a variety of neurodegenerative diseases including Alzheimer’s Disease, Parkinson’s Disease, and amyotrophic lateral sclerosis [10]. Furthermore, aggregation is an important consideration in the growing development of protein-based pharmaceuticals across manufacturing, efficacy, and safety viewpoints [11]. On the other hand, protein aggregates can also be functional [12], generating interest in development of amyloid-based biomaterials [13–15].

Given increasing recognition of the importance of protein aggregation in biology, significant research effort has been focused on enabling detailed structural and mechanistic understanding of the aggregation pathway. However, this is a particularly complex and challenging task given the heterogeneous and dynamic nature of the aggregation process. Many biophysical methods have been employed for the study of protein misfolding and aggregation [16]. Importantly, each approach has unique strengths and limitations, and a full picture can only be achieved by multiscale analysis using a combination of complementary approaches. In recent decades, MS has emerged as a particularly powerful tool for the study of protein structure, folding, and aggregation. As evidenced by countless examples in the literature, the diverse applications of MS methodology, together with an ability to couple to a variety of experimental approaches, have enabled new biophysical insight at almost every point of the protein aggregation pathway (Figure 1).

### Enabling features of MS in the study of protein aggregation

From the most fundamental perspective, an MS experiment involves the conversion of molecules into gas-phase ions, where they are separated in a mass analyzer according to their mass-to-charge ratios (*m/z*) [17]. For protein structural analysis, this technology was revolutionized with the advent of soft ionization techniques such as electrospray ionization (ESI), which enable large biomolecules to be carefully transferred into the gas phase while retaining solution phase relevance [18]. A number of key analytical features are critical to the impact of MS in the study of protein aggregation. For example, MS analysis is very sensitive and therefore does not require large amounts of protein. The measurements are also rapid, enabling time-resolved analysis of dynamic processes with structural resolution [19]. Perhaps most importantly, MS provides an ability to analyze species from within a heterogenous mixture without ensemble averaging, and can largely be carried out without altering the equilibrium conditions [20].

In the context of protein aggregation, if the protein/oligomer is enzymatically digested before MS analysis, then the resulting peptides can be separated according to their *m/z* as a basis of protein identification from biological samples. The measurement of molecular weight for intact proteins and their assemblies can enable the identification of stoichiometry to provide the number of monomeric units in an oligomeric structure. Furthermore, as native ESI-MS allows for preservation of noncovalent-binding interactions, the association of small-molecule cofactors, ligands, or chaperones with aggregating proteins may also be observed. Beyond simple molecular weight measurement however, extensive structural information can be provided through a range of coupled techniques, with key examples further described below. On this basis, MS has emerged as a highly sensitive technique for protein aggregation studies, offering complementary insights into the onset of amorphous aggregation and particularly amyloid fibril formation, including monomer and oligomer structural characterization, aggregation mechanisms, and inhibitor screening.

### Native ion mobility-MS

While native MS serves as a key method for the detection of intact protein assemblies, in cases where oligomeric complexes may overlap at the same *m/z* or a single species has different conformational states, multidimensional separation is critical. Ion mobility (IM) coupled to MS provides a means for further gas-phase separation of ions based on the rate at which they migrate through a neutral carrier gas under the influence of an external electric field. Importantly, the mobility of an ion can be related to rotationally averaged collision cross-section (CCS), and therefore can be used to infer information regarding biomolecular structure [21–23]. IM-MS therefore allows different conformational states of isobaric proteins and oligomers to be identified and monitored over time, and in response to varying perturbations.

### Tandem MS

Tandem MS allows selected ions to be isolated based on a first MS or IM analysis step, then submitted to activation and/or fragmentation. The resulting products are subsequently analysed to give MS/MS (or MS^n^ or ‘product-ion’) spectra, which contain rich structural information [24]. The resulting fragmentation patterns provide key information to assign a primary sequence, localize modifications, and in some cases, differentiate isomers and infer relative stability. Furthermore, certain fragmentation modes can localize accurately the interaction site of protein–protein and protein–ligand complexes by fragmenting the protein backbone while preserving the noncovalent bonding [25,26]. These features again provide broad novel insight into structural and mechanistic associations along the protein aggregation pathway.

### Protein footprinting

Observing the complexities of three-dimensional conformations and their dynamic interconversions on the protein aggregation pathway can also be enabled through an integrated MS-based approach commonly known as protein footprinting [27]. Here, different covalent labeling strategies are used to mark the solvent accessible surface area of proteins and reflect higher order structures. Such labeling approaches may be reversible, as is the case for hydrogen/deuterium exchange (HDX) in which a protein dissolved in D_2_O will exchange solvent-accessible and weakly H-bonded hydrogen atoms for deuterium in solution, with the increase in mass monitored as a function of time with sequence specificity to report such exchanges. HDX is typically employed for its ability to monitor protein folding/unfolding dynamics and locate regions involved in conformational changes [28]. Alternatively, irreversible protein-labeling reagents can react with solvent accessible amino acid side chains, leaving a permanent chemical marker that can be identified by standard proteomics methods. These analyses can also elucidate the binding location between protein–protein and protein–small-molecule interactions [27,29].

### Chemical cross-linking

Chemical cross-linking (CX) coupled with MS provides an additional method to characterize 3D conformations of proteins and identify protein-binding interfaces. In a basic CX-MS workflow, bifunctional small-molecule cross-linking reagents are used to covalently link two functional sites on the protein, typically with some defined reactivity toward specific amino acids. Since the links are made by a spacer with distinct length, localizing these linkage sites using proteomics methods provides an upper distance constraint to probe tertiary and quaternary structures [30–32]. As CX-MS is compatible with physiological conditions, it has been broadly applied for structural interrogation of protein aggregates.

### Quantitative proteome profiling

The advantages of MS that enable investigation of complex protein mixtures mean that it is increasingly being exploited to study structural and biophysical properties of proteins at a whole proteome scale. Of note, two additional MS approaches that probe proteome structural alterations *in situ* are based on limited proteolysis (LiP) and stability profiling. LiP-MS identifies structural changes in different cellular states or under different conditions by quantifying the relative sensitivity of proteins to a given protease through structure-dependent proteolytic patterns [33,34]. In contrast, thermal protein profiling reports on proteome-wide thermal stability by quantifying the soluble fraction of each protein at given points of a temperature gradient to yield a thermal denaturation profile [35,36]. In this way, cell lysates or even intact cells can be assessed for proteome-wide structural alterations, folding and stability, aggregation, and molecular interactions using MS data as a readout.

## Mass spectrometric insight at all stages of the aggregation pathway

Due to their dynamic, polydisperse, and polymorphic nature, aggregating proteins are particularly challenging to characterize using traditional structural biology methods. Here, recent studies are selected from among the many of this expansive field, to showcase the unique ability of MS in characterizing the underlying assembly pathway for a broad range of amyloidogenic and amorphously aggregating proteins. Furthermore, these studies outline the potential of MS as a tool for the screening of aggregation inhibitors and quality control monitoring.

### Monitoring early protein-misfolding events

The mechanisms by which proteins fold and misfold in solution remain the subject of significant study and debate. Disruption of native state(s) can occur because of many influences, and the early conformational changes from native structure have traditionally monitored in solution using a variety of techniques. However, IM-MS has proven invaluable to investigate the conformational landscape of many proteins [37,38] and to evaluate molecular dynamics in protein-folding studies [37,39,40]. In a recent example, IM-MS was applied to assess the misfolding pathway of bovine Cu/Zn-superoxide dismutase (SOD1), with dysregulation of this enzyme of significance in a range of neurodegenerative disorders. Through systematic denaturation of the holodimer by manipulation of solution conditions, elongation was first observed, followed by dissociation to holo-, single-metal, and finally extremely extended apo-monomeric SOD1 units, with loss of the metal cofactors found to be critical to destabilization [41].

**Figure 2:**
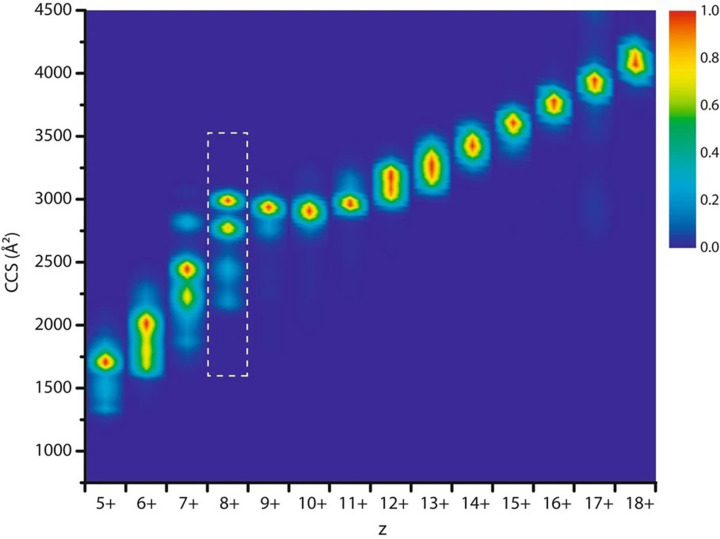
CCS profiles from IM-MS experiments comparing conformational ensembles for the 8+ charge state of α synuclein The metal salts all lead to compaction; however, the conformational profile is characteristic for each metal salt, suggesting that counterions also play an important role. Figure reproduced under a creative Commons CC BY Attribution 4.0 International License from [44].

A range of IM-MS studies have focused on early conformational changes of amyloidogenic proteins, showing a structural reorganization from extended structures of the intrinsically disordered proteins clinically implicated in neurodegenerative disorders to more compact states which precede aggregation [42,43]. Similarly, native IM-MS was recently used to characterize heterogeneous interactions of metal ions and their global structural effects on α-synuclein [44]. Binding stoichiometries of a variety of metal ions and their affinity for the intrinsically disordered protein were determined, and the effect of these interactors on the conformational space of the protein was shown to depend not only on their charge but also their chemical nature (Figure 2).

Pulse-labeling HDX-MS also enables direct monitoring of rapid events such as the earliest stages of protein misfolding, with high structural and temporal resolution. For example, this technique has provided enhanced understanding of the mechanism of aggregation for the clinically important proteins amyloid-β, α-synuclein, and tau [45–48]. By monitoring the structure of these intrinsically disordered proteins during aggregation, it was possible to probe kinetic intermediates along the aggregation pathway, and understand how the appearance of early structural features contribute to the initiation and progression of the aggregation cascade at critical stages, including in membrane environments [49].

### From monomers to soluble oligomers

Growing evidence suggests that the soluble oligomers on pathway to large aggregates are primary contributors to cell toxicity, particularly in the context of the diverse range of amyloid disorders [50]. Once again, the heterogeneous qualities and often low abundance of these intermediates make them difficult to characterize, particularly where ensemble-averaged information is misleading, and hence IM-MS has featured heavily in the study of soluble oligomeric intermediates. Early reports demonstrated the assembly of amyloidogenic fragments such as the peptide NNQQNY and that derived from the human plasma protein transthyretin (TTR_105–115_), describing oligomeric states and aggregation over time. For example, the NNQQNY peptide was shown to form extended, likely β-sheet-rich structures, for oligomers beyond octamers, representing a distinct transition toward the amyloid pathway from that point [51]. In comparison, the TTR fragment showed that dimeric and tetrameric species persist more stably, with the tetramer suggested to be a key on-pathway oligomer [52]. Amyloid-β oligomers have also been extensively characterized in similar studies, with the difference in aggregation propensity of Aβ40 and Aβ42 peptides explained by IM-MS-derived structural data, based on their ability to form proposed toxic dodecameric intermediates [53]. One suggested mechanism for toxicity of amyloid-β oligomeric intermediates is the assembly of these in biological membranes to form pores that induce cell leakage. Direct detection of such putative assemblies is similarly complicated by a high level of heterogeneity; however, this hypothesis has been supported by native MS with observation of Aβ42 oligomerization in a micellar environment, forming hexamers with CCSs in agreement with a β-barrel pore [54].

Kinetic-pulsed variations of HDX have also been used to characterize early amyloidogenic intermediates [55], enabling structural insights into the aggregates populated during the fibril-forming process, and providing kinetic and mechanistic information regarding the structural reorganizations that take place during aggregation. Furthermore, as an example of the utility of covalent footprinting approaches, hydroxyl radical-based fast photochemical oxidation of proteins (FPOP) and MS was recently applied to monitor the time-course of Aβ1–42 aggregation. This provided insight into time-dependent transitions of monomers and paranuclei through to fibrillar aggregates by allowing the conformational changes to be mapped at subregional and even amino-acid-residue levels [56]. With the dynamic interconversion of oligomeric species highlighted in such studies, molecular stability can be gained through CX (and the formation of covalent bonds) between adjacent species. This approach was recently demonstrated for amyloid-β oligomers by stabilizing with a bifunctional linker that traps the peptide in soluble oligomeric conformations in the 50–300 kDa range as characterized by native top-down MS [57]. Notably, these cross-linked species were particularly potent in inducing negative biological effects, supporting the utility of CX-MS as a tool in aggregation structure–function studies.

### Mature amyloid fibrils

Given extensive characterization by X-ray crystallography, nuclear magnetic spectroscopy and, more recently, cryoelectron microscopy, the structure of mature amyloid fibrils is largely well understood. Nevertheless, while gas-phase analysis of amyloid fibrils is particularly challenging due to their enormous size, MS is still able to further probe the properties of such mature assemblies. For example, molecular-weight determination is the most direct way of determining the aggregation state of a protein. Recent applications of charge-detection MS, a single-molecule method where the mass of each ion is directly determined from individual measurements of *m/z* and charge [58], demonstrated an ability to detect and characterize the heterogeneity and the polymorphism of fibril populations on the order of 100–400 MDa [59,60]. This offers future opportunities to obtain kinetic growth profiles of very large aggregates. HDX-MS also serves as an additional approach to yield dynamic information for mature protein aggregates. Of note, amyloid fibrils have been shown not to exist as static assemblies, but instead are dynamic complexes from which monomer units ‘recycle’ through dynamic dissociation and association at fibril ends [61,62]. As this occurs on a biologically relevant time scale, mature fibrils may in fact not be a protective sink for misfolded protein as is sometimes proposed, but instead must still be considered as a potential source of toxic oligomers [63].

### Aggregation inhibitors

A range of MS-based methods are able to observe and interrogate binding interactions of protein–ligand complexes [64]. Often, this can be achieved concomitantly with monitoring changes in relative abundances and distributions of particular conformations or oligomeric states. Consequently, correlating ligand binding with structural changes and alterations in aggregation propensity provides a means to screen and identify novel inhibitory compounds. Given the rapid and sensitive detection of ligand binding by MS, the technology lends itself nicely to high-throughput screening, with on the order of 1000 molecules possibly screened per milligram of protein, at less than 1 min per sample [65]. Many compounds have undergone MS-based analysis to validate their antiaggregatory properties. These range from small molecules [65–67] and peptides [68–71] to large chaperones [72–74]. Notably, in contrast with many other approaches, it is possible to use MS-based screening to identify the specific conformational states to which inhibitors bind [75,76] as well as to reveal the mode of action and binding affinities/competitive interactions of inhibitors by analysis of the resulting spectra [65]. Perhaps, the most extensive high-throughput screen to date based on IM-MS analysis from a 96-well plate format classified a variety of small molecules as potential inhibitors of human islet amyloid polypeptide aggregation (hIAPP) or amyloid-β aggregation as specific, nonspecific, or colloidal binders and was able to discover a new inhibitor of hIAPP amyloid assembly [65].

### Biopharma

**Figure 3:**
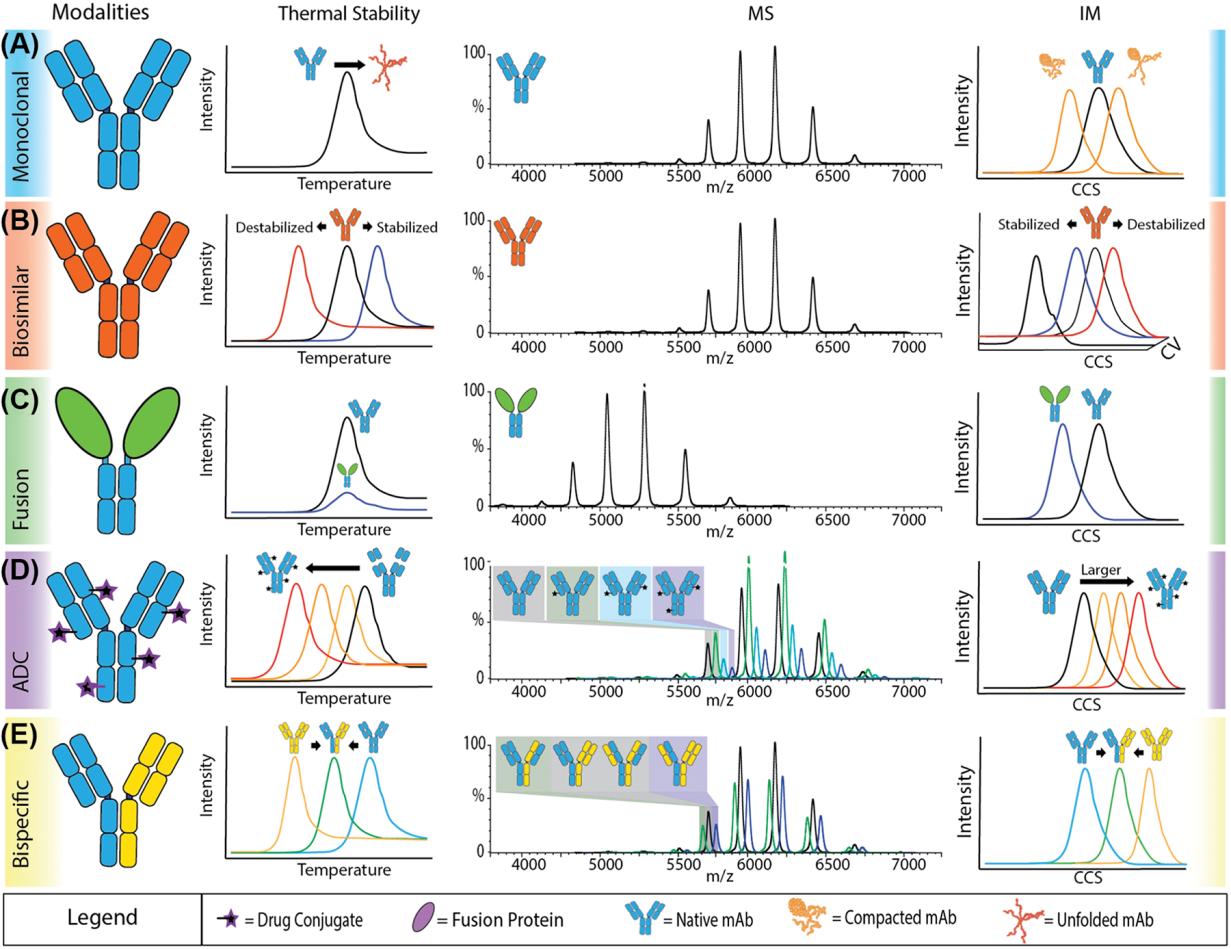
MS-based datasets for stability and aggregation analysis of biotherapeutic modalities Thermal protein-profiling experiments report shifts to lower Tm values, indicating a decrease in stability while higher values indicate an increase in stability. Changes in mass measurements can indicate different covalent structures or stoichiometries. Changes to CCS values recorded by IM indicate either more compact structures or larger, often unfolded structures. By applying gas-phase activation energy and monitoring unfolding, relative stability can be observed. Figure reprinted with permission from [78]. Copyright (2022) American Chemical Society.

While much focus in the protein aggregation field has been directed toward amyloid aggregation due to impacts in human health, amorphous aggregation is also of significant note in a biopharmaceutical context. Here, aggregates must be strictly controlled as they are not only less efficacious but resultant particulates may cause serious immunological responses when administered [77]. Liquid chromatography-MS-based methods are already widely embedded in the pharmaceutical industry within multiattribute monitoring workflows; however, structurally sensitive MS methods present a growth area for the rapid analysis of biopharmaceuticals at the higher-order structure level. Recent advancements in MS techniques have demonstrated broad potential for characterization of monoclonal antibodies (mAbs) and biosimilars by monitoring of higher-order interactions, aggregation, degradation, and stability [78]. For example, as well as successful application to epitope mapping, covalent footprinting has been used to probe local and long-range conformational changes in mAbs associated with reversible self-association and aggregation propensity [79]. Gas-phase collisional activation followed by IM-MS, now commonly referred to as collision-induced unfolding (CIU), has also been used to provide rapid assessment of protein stability [80], including for a range of antibody–drug conjugates to demonstrate stabilization of monoclonal antibodies through drug conjugation [81]. Given the diverse information content that can be afforded on protein stability and aggregation through MS- and IM-based methods (Figure 3), this technology is well placed to become a standard component of future analytical workflows for quality control and similarity assessment of intact protein therapeutics.

## Conclusions and future outlook

Although there are many different approaches to study protein aggregation, MS has firmly established itself as critical to this pursuit based on the diversity of information afforded. It is clear from the proliferation of protein aggregation studies reported in recent decades, which employ MS methodologies that it can reveal many previously hidden aspects of protein misfolding and aggregation across the entire structural landscape. Importantly, a key strength of MS is its ability to further combine with orthogonal techniques. Continued advancements in MS technology and an ongoing drive toward integrated structural biology, where multiple methods combine to contribute to a deep understanding of protein structure and dynamics, will lead to a rich knowledge of the fundamental factors that modulate aggregation. We might ultimately see MS-driven technologies routinely describe protein aggregation states across the whole proteome and under a wide diversity of conditions, providing further opportunities to intervene to the advantage of human health and biotechnology.

## Summary

- Protein aggregation occurs via a complex, multistep pathway involving aberrant assembly of misfolded proteins.
- MS offers unique advantages for the biophysical analysis of heterogeneous and dynamic processes involved in protein misfolding and aggregation.
- Utilizing MS-based detection with various experimental approaches, information regarding structural changes, stability, self-assembly, and other molecular interactions and dynamics can be captured along the entire protein aggregation pathway.
- MS-based understanding of protein aggregation can have broad and impact across areas of health and biotechnology.

## Open Access

Open access for the present article was enabled by the participation of University Of Adelaide in an all-inclusive *Read & Publish* agreement with Portland Press and the Biochemical Society under a transformative agreement with CAUL.

## Abbreviations

CCS: Collision cross-section
CX: Chemical cross-linking
ESI: electrospray ionization
FPOP: fast photochemical oxidation of protein
HDX: hydrogen/deuterium exchange
hIAPP: human islet amyloid polypeptide
IM: ion mobility
LiP: limited proteolysis
mAbs: monoclonal antibodies
MS: mass spectrometry
m/z: mass-to-charge ratio
SOD: superoxide dismutase
TTR: transthyretin
